# Integrative Bioinformatics Analysis of hsa-miR-21 in Breast Cancer Reveals a Prognostic Hub-Gene Signature

**DOI:** 10.3390/ijms27020865

**Published:** 2026-01-15

**Authors:** Maria Rosaria Tumolo, Luana Conte, Roberto Guarino, Ugo De Giorgi, Elisabetta De Matteis, Saverio Sabina

**Affiliations:** 1 Institute for Research on Population & Social Policies, Research Unit of Brindisi, National Research Council, 72100 Brindisi, Italy; 2 Department of Physics and Chemistry “E. Segrè”, University of Palermo, 90128 Palermo, Italy; luana.conte@unipa.it; 3 Advanced Data Analysis in Medicine (ADAM), Laboratory of Interdisciplinary Research Applied to Medicine (DReAM), Local Health Authority (ASL) of Lecce, 73100 Lecce, Italy; 4 Institute of Clinical Physiology, Branch of Lecce, National Research Council, 73100 Lecce, Italy; roberto.guarino@cnr.it (R.G.); saverio.sabina@cnr.it (S.S.); 5 Department of Experimental Medicine, University of Salento, 73100 Lecce, Italy; ugo.degiorgi@unisalento.it; 6 Oncology Unit, ‘Vito Fazzi’ Hospital, 73100 Lecce, Italy; dr.dematteis.elisabetta@gmail.com

**Keywords:** miR-21, bioinformatic analysis, hub genes, breast cancer, molecular pathways

## Abstract

Breast cancer (BC) is the most frequently diagnosed malignancy in women and remains a leading cause of cancer-related mortality worldwide. Among the oncogenic microRNAs, hsa-miR-21 has been consistently implicated in tumorigenesis, yet a comprehensive network-level understanding of its regulatory landscape in BC is lacking. In this study, we performed an integrative bioinformatics analysis to characterize the molecular pathways and prognostic impact of hsa-miR-21. Experimentally validated mRNA targets were retrieved from miRTarBase and used to construct a high-confidence protein–protein interaction network via STRING, followed by hub-gene prioritization in Cytoscape. Functional enrichment analyses were conducted with DAVID to assess Gene Ontology (GO) categories and KEGG pathways. Survival analyses were performed in large BC cohorts from METABRIC and TCGA using the Kaplan–Meier Plotter. We identified 12 hub genes that are central regulators of apoptosis, proliferation, immune signaling, and transcriptional control. GO and KEGG analyses revealed enrichment in cancer-related, immune, and metabolic pathways, underscoring the pleiotropic role of miR-21. While miR-21 expression alone was not significantly associated with overall survival, a composite hub-gene signature demonstrated strong prognostic value. These findings highlight the importance of network-level biomarkers in BC and provide a reproducible framework for dissecting the clinical relevance of disease-associated miRNAs.

## 1. Introduction

Breast cancer (BC) is the most frequently diagnosed cancer in women and remains a leading cause of cancer-related mortality worldwide [1]. Its pronounced molecular heterogeneity, encompassing diverse histological subtypes and genomic alterations, poses a major challenge for accurate diagnosis, prognosis, and treatment [2].

In recent decades, small non-coding RNAs, particularly microRNAs (miRNAs), have emerged as critical post-transcriptional regulators of gene expression [3]. By binding to the 3′ untranslated regions of target messenger RNAs, miRNAs influence mRNA stability and translation, thereby modulating fundamental biological processes such as cell proliferation, apoptosis, metastasis, and therapeutic response [4]. Each miRNA can regulate multiple target genes, while distinct miRNAs often converge on shared pathways, creating intricate regulatory networks [5]. It is estimated that nearly one-third of human genes are under miRNA regulation, underscoring their pervasive role in cellular homeostasis [6].

Among cancer-associated miRNAs, hsa-miR-21 consistently emerges as one of the most prominent oncogenic miRNAs, or “oncomiRs” [7]. Its overexpression has been reported in various malignancies [8], including BC [9,10]. Individual targets such as PTEN, PDCD4, and TPM1 have been characterized, indicating that its oncogenic potential derives from the coordinated suppression of multiple tumor-suppressive pathways [11,12]. However, most studies have examined isolated targets, leaving the broader network-level impact of miR-21 in BC largely unresolved [13,14].

miR-21 gives rise to two mature strands, miR-21-5p (guide strand) and miR-21-3p (passenger strand). Both isoforms have been shown to target tumor suppressor genes and contribute to oncogenic signaling [15,16], highlighting the need for analyses that capture their combined regulatory influence.

Traditional approaches have primarily focused on single miRNA–mRNA interactions, yielding valuable but fragmented insights into miR-21 biology [17]. In contrast, bioinformatics methods enable a systems-level perspective [18,19,20]. By integrating experimentally validated interactions with protein–protein interaction (PPI) networks and functional enrichment analyses, such approaches can reconstruct regulatory circuits and signaling pathways, facilitating the identification of hub genes with prognostic and therapeutic relevance. Moreover, reproducible computational pipelines can be readily extended to other disease-associated miRNAs, increasing their translational value.

Although several previous studies have explored miR-21–related networks in BC, most relied on single datasets or limited network metrics, providing partial or non-reproducible views of its regulatory landscape. In this work, we combine multiple large-scale breast cancer datasets (TCGA, METABRIC, and GEO) and apply eight independent topological algorithms to derive a consensus set of hub genes consistently ranked across methods. This consensus-based strategy enhances robustness and reproducibility compared with earlier analyses and directly links network-level findings to clinical outcomes through integrated survival analysis.

Here, we present an integrative bioinformatics analysis of hsa-miR-21 in BC, focusing on high-confidence, experimentally supported mRNA targets. Through PPI network construction, hub-gene prioritization using multiple topological algorithms, and Gene Ontology (GO) and Kyoto Encyclopedia of Genes and Genomes (KEGG) enrichment analyses, we delineated central molecular nodes and pathways modulated by miR-21. Finally, survival analyses in large BC cohorts were performed to evaluate the prognostic relevance of the identified hub-gene signature. This comprehensive strategy provides novel insights into the regulatory landscape of miR-21 and establishes a replicable bioinformatics framework with direct implications for prognostic biomarker discovery.

## 2. Results

### 2.1. Identification of miR-21 Targets and Functional Analysis

From miRTarBase, 154 experimentally validated target genes were retrieved for hsa-miR-21-5p and 33 for hsa-miR-21-3p. After filtering for high-confidence interactions in STRING, the protein–protein interaction (PPI) network consisted of 163 nodes and 276 edges.

**Figure 1:**
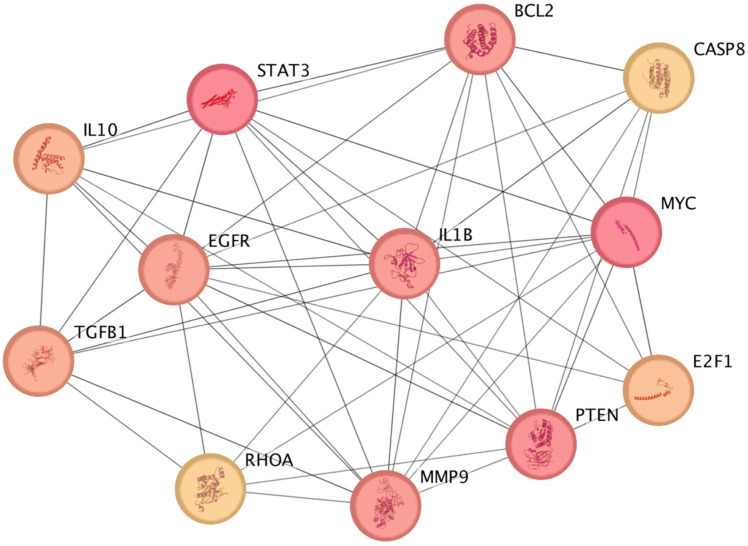
Identification of 12 hub genes determined through 8 algorithms in CytoHubba. Node color intensity reflects the degree of connectivity, with darker nodes representing genes with higher degree values (i.e., more network interactions).

Using CytoHubba in Cytoscape, 12 hub genes (*STAT3*, *MYC*, *PTEN*, *BCL2*, *IL1B*, *MMP9*, *EGFR*, *TGFB1*, *IL10*, *E2F1*, *CASP8*, and *RHOA*) were identified as top-ranked by all eight topological algorithms, indicating strong centrality and potential biological importance (Table 1, Figure 1).

**Figure 2:**
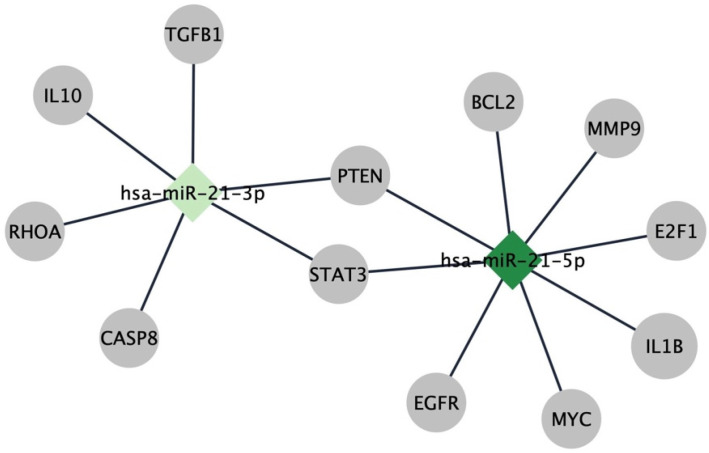
Interaction network illustrating the relationship between the miRNAs and the 12 identified hub genes. Diamonds represent miRNAs, while ellipses represent the corresponding target genes. A darker color (dark green) indicates a higher connectivity within the network.

The regulatory network linking miR-21-5p/3p to these hub genes is shown in Figure 2, highlighting key post-transcriptional interactions with potential relevance to Breast Cancer (BC) progression and prognosis.

**Figure 3:**
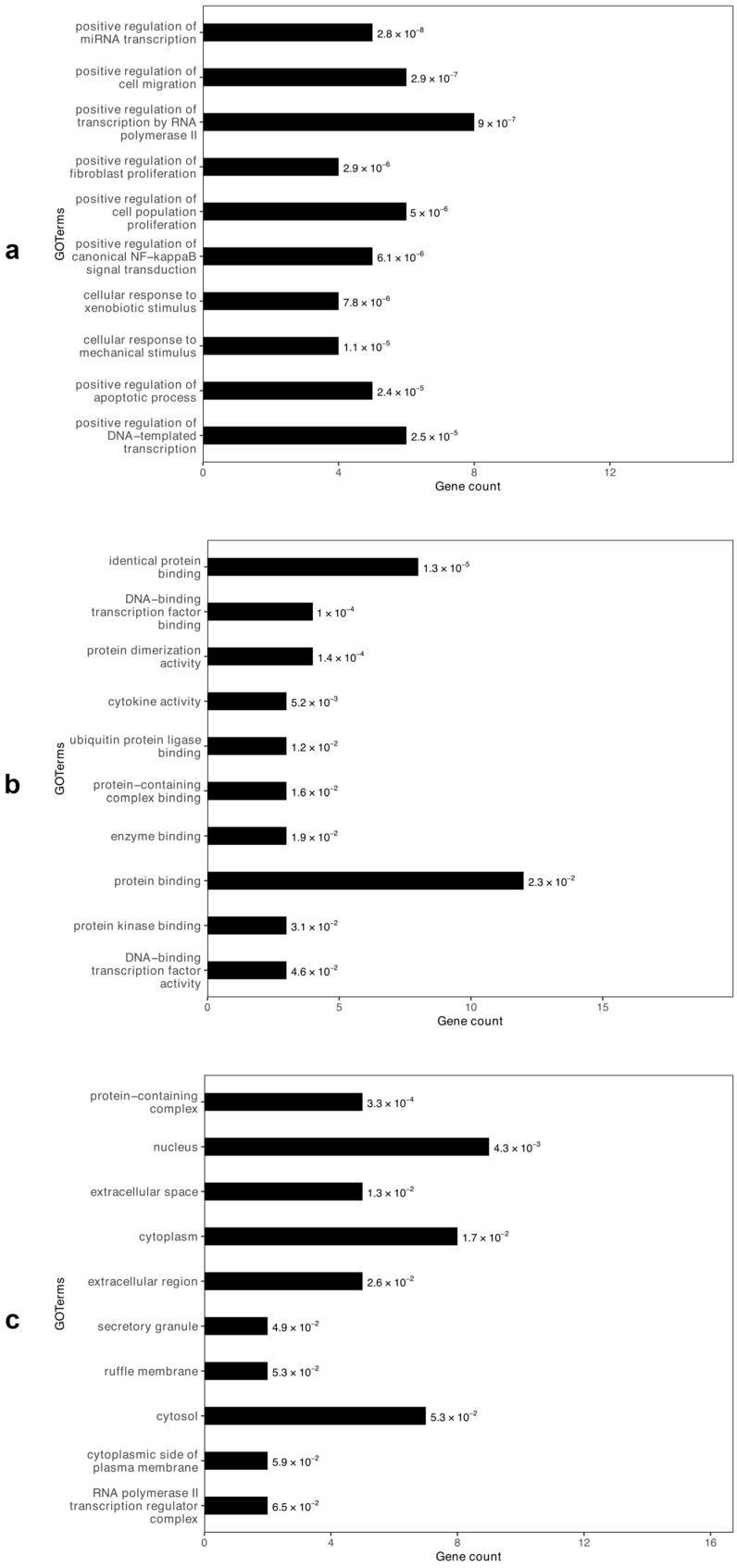
Gene Ontology (GO) analysis of the miRNA hub genes: (**a**) Biological Processes, (**b**) Molecular Functions, and (**c**) Cellular Components. The GO terms are arranged according to their *p*-values, in descending order. Numerical values displayed beside each bar indicate the corresponding *p*-values.

Gene Ontology (GO) enrichment analysis revealed that the hub genes were mainly involved in biological processes (BP) related to cell proliferation and migration, transcriptional regulation (including RNA polymerase II–dependent transcription), apoptosis, NF-κB signaling, and cellular stress responses (Figure 3a). In the Molecular Function (MF) category, significant terms included transcription factor binding, cytokine activity, protein dimerization, and protein kinase binding (Figure 3b). The Cellular Component (CC) analysis indicated predominant localization in the nucleus, cytoplasm, cytosol, extracellular space, and ruffle membrane (Figure 3c) (Table S1).

**Figure 4:**
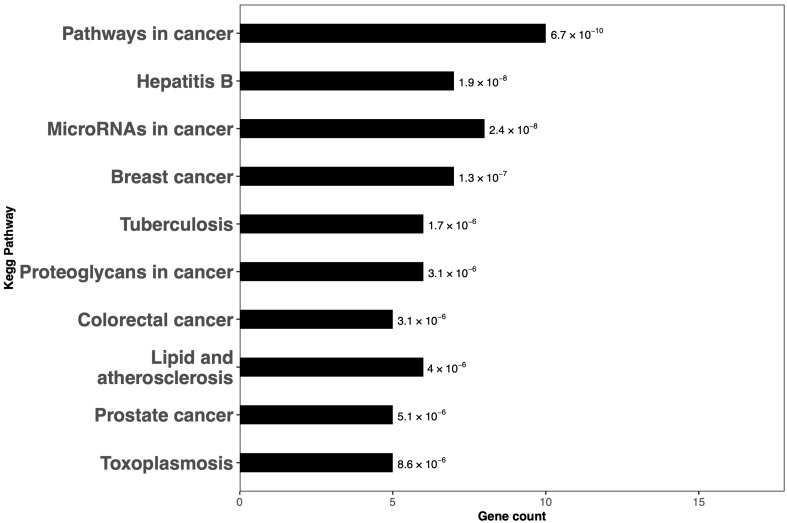
KEGG pathway enrichment analysis of the miRNA hub genes. The pathways are arranged according to their *p*-values, in descending order. Numerical values displayed beside each bar indicate the corresponding *p*-values.

KEGG pathway enrichment analysis identified the top 10 enriched pathways, which were primarily cancer-related (Pathways in cancer, MicroRNAs in cancer, Breast cancer, Colorectal cancer, Prostate cancer, Proteoglycans in cancer), alongside pathways associated with infectious diseases (Hepatitis B, Tuberculosis, Toxoplasmosis) and cardiovascular conditions (Lipid and atherosclerosis) (Figure 4) (Table S2).

### 2.2. Survival Analysis of miR-21 and Composite Hub-Gene Signature

Kaplan–Meier analysis showed no significant association between miR-21 expression and overall survival (OS) in either the METABRIC cohort (*n* = 1262; HR = 1.10, 95% CI = 0.90–1.34, log-rank *p* = 0.35; Supplementary Figure S1) or the TCGA-BRCA dataset (*n* = 1078; HR = 1.18, 95% CI = 0.85–1.63, log-rank *p* = 0.32; Supplementary Figure S2).

**Figure 5:**
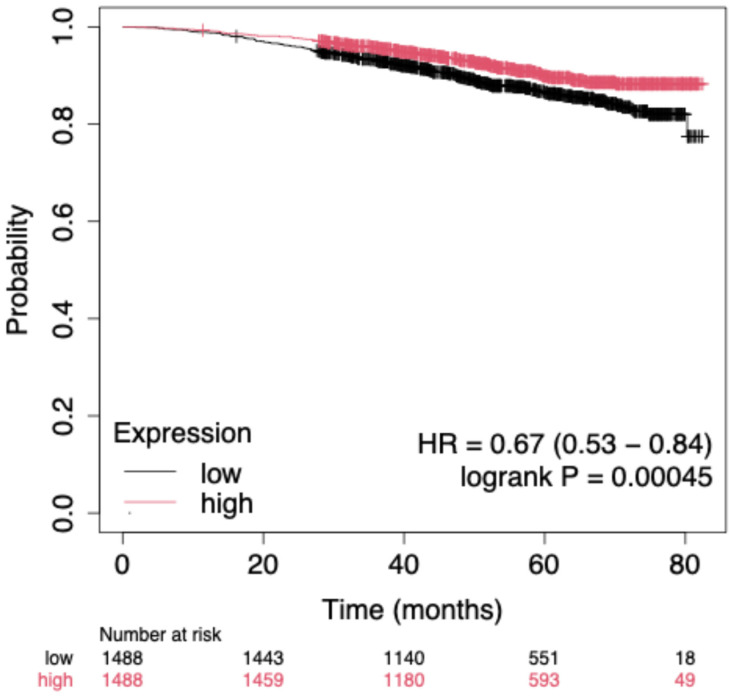
Kaplan–Meier survival curve for the miR-21 hub-gene signature in the integrated “All breast cancer” cohort (Kaplan–Meier Plotter), including TCGA, METABRIC, and GEO datasets (*n* = 2976). Patients were divided into high- and low-expression groups based on the median of the mean normalized expression values of the 12 hub genes.

In contrast, a composite hub-gene signature derived from the top regulatory targets (*STAT3*, *BCL2*, *MYC*, *EGFR*, *MMP9*, *TGFB1*, *IL1B*, *IL10*, *E2F1*, *RHOA*, *PTEN*, and *CASP8*) demonstrated a significant prognostic effect. Patients with high signature expression exhibited significantly improved OS compared with those with low expression (HR = 0.67, 95% CI = 0.53–0.84, log-rank *p* = 4.5 × 10^−4^; Figure 5). These findings underscore the prognostic value of the miR-21–regulated molecular network and suggest that integrative multi-gene signatures may provide stronger clinical insights than single-miRNA analyses.

The prognostic performance of the 12-gene hub signature was further evaluated within major BC. The signature remained significantly associated with improved OS in the ER-positive (*n* = 2.575, *p* = 0.0408) and Basal-like (PAM50, *n* = 309, *p* = 0.0342) subgroups, whereas non-significant trends were observed in the Luminal A (*n* = 1.504, *p* = 0.2866) and HER2-enriched (*n* = 295, *p* = 0.1395) cohorts (Supplementary Figures S3–S6). These findings indicate that the prognostic value of the composite signature extends beyond the global cohort and is particularly evident in ER-positive and Basal-like breast cancers.

## 3. Discussion

Gaining insight into the molecular mechanisms driving Breast Cancer (BC) onset, progression, and dissemination is fundamental for improving prognostic assessment and therapeutic approaches. MiRNAs exert their biological functions primarily by modulating target gene expression at the post-transcriptional level [21]. Consequently, computational prediction of miRNA targets represents a key step toward unraveling the molecular basis of tumor development [22].

Although miR-21 has been extensively investigated in BC [7], most previous network analyses were limited to individual datasets or single topological metrics, providing a fragmented and sometimes non-reproducible view of its regulatory landscape. In contrast, our study integrates multiple large-scale transcriptomic cohorts (TCGA, METABRIC, and GEO) and applies eight independent topological algorithms to derive a consensus set of hub genes that remain consistently prioritized across methods. This consensus-based approach enhances robustness and reproducibility compared with earlier studies and allows direct linkage of network topology to patient survival. In this sense, the present work represents an advancement over prior miR-21 analyses by combining methodological rigor with clinical validation in a large multi-cohort context.

By integrating experimentally validated targets from miRTarBase with protein–protein interaction (PPI) network construction and topological prioritization, we identified 12 hub genes (*STAT3*, *MYC*, *PTEN*, *BCL2*, *IL1B*, *MMP9*, *EGFR*, *TGFB1*, *IL10*, *E2F1*, *CASP8*, *RHOA*). These genes are central regulators of apoptosis, proliferation, immune modulation, and oncogenic transcriptional programs. For example, *PTEN* and *CASP8* act as tumor suppressors [23], whereas *STAT3*, *MYC*, *EGFR*, and *TGFB1* are canonical oncogenic drivers implicated in cell cycle progression, stemness, and therapy resistance, particularly in triple-negative BC [24,25].

Gene Ontology (GO) enrichment analyses further substantiated the biological relevance of these hub genes, revealing enrichment in transcriptional regulation, cell migration, fibroblast proliferation, NF-κB signaling, apoptosis, and stress responses [26,27,28]. These findings support the multifaceted role of miR-21 in promoting tumor growth, invasiveness, and adaptation to hostile microenvironmental conditions. Similarly, KEGG pathway analysis highlighted not only canonical cancer pathways but also infectious and cardiovascular pathways, suggesting that miR-21 target networks intersect with immune and metabolic processes. Such pleiotropic regulation aligns with the emerging recognition of miR-21 as a master regulator that orchestrates cancer hallmarks at multiple levels, from intracellular signaling to tumor–stroma interactions [29,30,31]. Compared with previous studies that mainly described isolated functional categories, our integrative approach uncovered a convergent enrichment of immune-related and metabolic pathways, indicating that miR-21 may act as a molecular bridge between tumor immunoregulation and cellular metabolism. This intersection, often overlooked in earlier analyses, points to a potential systemic role of miR-21 beyond canonical oncogenic signaling [9,16].

Interestingly, while miR-21 expression alone did not associate with overall survival in large patient cohorts (METABRIC, TCGA), a composite hub-gene signature derived from miR-21 targets showed a robust and statistically significant prognostic effect. Patients with high expression of the signature had significantly better overall survival.

Although several hub genes included in the signature (e.g., *STAT3*, *MYC*, *EGFR*) are classically described as oncogenic drivers [13,14], their aggregated expression correlated with improved survival. This apparently paradoxical association likely reflects a combination of biological and analytical factors. First, the integrated Kaplan–Meier Plotter dataset is predominantly composed of ER-positive and Luminal A tumors, which generally have more favorable outcomes and may show subtype-specific expression patterns of these genes. Second, the hub-gene signature captures both oncogenic and tumor-suppressive targets (such as *PTEN* and *CASP8*), and the balance between these components may influence the overall prognostic trend. Third, residual batch effects or normalization differences among the combined cohorts may contribute to signal attenuation or inversion in multi-cohort analyses. Importantly, the positive prognostic association was consistent across molecular subtypes, supporting the robustness of the finding despite potential context-dependent effects.

While experimental validation was beyond the scope of this bioinformatics study, all identified hub genes are supported by strong experimental evidence in miRTarBase and have established roles in BC signaling. Future work will aim to validate the prognostic hub-gene signature in external datasets and functional models to confirm its biological and clinical significance.

From a translational perspective, these results have several implications. First, multi-gene signatures derived from miRNA target networks may serve as surrogate readouts of miRNA activity, offering improved prognostic stratification in BC. Second, several of the identified hub genes (e.g., *EGFR*, *STAT3*, *TGFB1*) are druggable targets [32,33,34,35,36], raising the possibility that miR-21–regulated networks could inform therapeutic decision-making. Third, the integrative pipeline employed here—combining experimentally validated interactions, multi-algorithm hub scoring, and survival validation—provides a reproducible framework that can be generalized to other miRNAs and disease contexts, addressing the need for methodological transparency and reproducibility in network-based studies.

Our study has some limitations. First, it relies on publicly available interaction databases and bulk transcriptomic data, which may not fully capture context-specific or cell type–specific effects. Second, the identification of hub genes depends on network topology and on the choice of scoring algorithms in CytoHubba, which may introduce methodological bias. Third, no experimental validation was performed to directly confirm the regulatory interactions or prognostic relevance of the hub-gene signature. In addition, integrating other omics layers—such as methylation, proteomic, or phosphoproteomic data—could refine the understanding of miR-21–mediated regulatory networks and better capture their multi-level effects. The Kaplan–Meier Plotter database used for survival analyses provides only overall survival data for BC, limiting evaluation of disease-specific or progression-free survival; future studies should therefore test the signature against additional clinical endpoints to further assess its prognostic robustness. Experimental validation through functional assays in vitro and in vivo, together with independent patient cohorts, will be essential to confirm the biological and clinical relevance of the findings.

Moreover, integrating single-cell and spatial transcriptomics could further dissect the contribution of tumor, stromal, and immune compartments to miR-21–mediated networks. Prospective clinical studies are needed to test whether the identified hub-gene signature can be used as a biomarker for risk stratification or for predicting response to targeted therapies, particularly inhibitors of *EGFR*, *STAT3*, and *TGFB1*. Finally, extending this bioinformatics framework to other oncogenic miRNAs may uncover additional prognostic signatures and therapeutic opportunities across different malignancies.

## 4. Materials and Methods

### 4.1. PPI Network Analysis and Hub Gene Prioritization of miR-21 Targets

Experimentally validated mRNA targets of hsa-miR-21 were retrieved from miRTarBase (https://bio.tools/mirtarbase, accessed on 20 July 2025), including only interactions supported by robust experimental evidence such as reporter assays, Western blotting, or qPCR [37]. Validation data in miRTarBase originate from diverse human cell lines and tissues; no further restriction to specific cellular models was applied, as the analysis focused on experimentally confirmed miRNA–target interactions irrespective of the original validation system.

The resulting gene set was used to construct a protein–protein interaction (PPI) network through the STRING database (http://www.string-db.org/, accessed on 25 July 2025), restricting the analysis to high-confidence interactions (combined score ≥ 0.700) [38]. The interaction network was imported into Cytoscape (version 3.10.2) for visualization and downstream analyses [39].

Hub genes were identified using the CytoHubba plugin in Cytoscape by applying eight topological algorithms: Degree (ranks nodes by the number of direct connections), Maximum Clique Centrality (MCC) (identifies nodes involved in multiple maximal cliques), Closeness (measures how close a node is to all others in the network, based on average shortest path length), Maximum Neighborhood Component (MNC) (quantifies the size of the largest connected component in each node’s neighborhood), Stress (counts the number of shortest paths passing through a node), Betweenness (assesses how often a node lies on the shortest paths between other nodes), Bottleneck (identifies nodes that control the main information flow between sub-networks), and Edge Percolated Component (EPC) (evaluates node robustness under edge percolation, identifying stable hubs) [40]. For each algorithm, the top-ranked nodes were selected, and consensus hub genes were defined as those appearing among the top candidates across all eight algorithms, indicating consistent centrality by multiple independent topological criteria [40]. As CytoHubba does not provide statistical significance or *p*-values, consistency across algorithms was used as the main criterion of robustness.

### 4.2. Functional Enrichment Analysis

Functional enrichment analysis of the top hub genes was performed using DAVID (Database for Annotation, Visualization and Integrated Discovery, v6.8) [41]. Enrichment was assessed for Gene Ontology (GO) categories—Biological Process (BP), Molecular Function (MF), and Cellular Component (CC) [42]—as well as for Kyoto Encyclopedia of Genes and Genomes (KEGG) pathways [43]. Terms and pathways with *p* < 0.05 after Benjamini–Hochberg False Discovery Rate (FDR) correction were considered statistically significant.

### 4.3. Survival Analysis

To evaluate the prognostic significance of miR-21–associated networks, survival analysis was conducted using the Kaplan–Meier Plotter (https://kmplot.com/analysis/ (accessed on 27 July 2025)) [44,45], which integrates gene expression and clinical outcome data from large breast cancer cohorts including METABRIC and TCGA (https://doi.org/10.1016/j.csbj.2021.07.014).

Overall survival (OS) was selected as the primary clinical endpoint, and all analyses were conducted following the platform’s default configuration. For single-cohort evaluations, the TCGA (RNA-seq, FPKM-normalized) and METABRIC (microarray, MAS5-normalized) datasets were analyzed separately. For multi-gene analyses, the “Use mean expression of selected genes” option was applied, so that the composite hub-gene signature represented the arithmetic mean of the normalized (log_2_-scaled) expression values provided by the database for the selected genes. No additional weighting based on reported oncogenic or tumor-suppressive roles was applied. Patients were dichotomized into high and low expression groups using the median value as cutoff (“Split patients by median”), with the “Censor at threshold” and “Compute median survival” options enabled.

The survival analyses were conducted using the “All breast cancer” integrated dataset available in the Kaplan–Meier Plotter, which combines TCGA, METABRIC, and GEO breast cancer cohorts (*n* = 2.976) after normalization and batch correction. To further assess the clinical robustness of the 12-gene hub signature, subgroup analyses were performed using the Kaplan–Meier Plotter “Restrict analysis to subtypes” option. Survival analyses were repeated within major molecular and clinical subgroups, including ER-positive, Basal-like (PAM50), Luminal A (PAM50), and HER2-enriched (PAM50) tumors.

Hazard ratios (HRs) and 95% confidence intervals (CIs) were estimated using univariate Cox regression as implemented in the platform, and log-rank *p*-values were calculated to assess statistical significance.

## 5. Conclusions

Taken together, our results emphasize the importance of moving beyond reductionist approaches and adopting integrative strategies that capture the complexity of miRNA–mRNA regulatory networks. Through the integration of multiple Breast Cancer cohorts and independent topological algorithms, our study refines current models of miR-21 regulation and establishes a reproducible foundation for network-based biomarker discovery. The identification of a prognostic hub-gene signature downstream of miR-21 further demonstrates the translational potential of such systems-level approaches in precision oncology.

## Figures and Tables

**Table 1:** Top 20 ranked hub genes using CytoHubba.

Degree	Bottleneck	MCC	Stress	Closeness	EPC	MNC	Betweenness	Overlap
*STAT3*	*MYC*	*STAT3*	*PTEN*	*MYC*	*STAT3*	*STAT3*	*MYC*	*STAT3*
*MYC*	*EGFR*	*IL1B*	*TGFB1*	*STAT3*	*PTEN*	*MYC*	*PTEN*	*MYC*
*PTEN*	*TGFB1*	*MMP9*	*MYC*	*PTEN*	*IL1B*	*PTEN*	*MMP9*	*PTEN*
*BCL2*	*MMP9*	*PTEN*	*STAT3*	*EGFR*	*MMP9*	*IL1B*	*TGFB1*	*BCL2*
*IL1B*	*STAT3*	*BCL2*	*MMP9*	*IL1B*	*MYC*	*BCL2*	*STAT3*	
*MMP9*	*PTEN*	*EGFR*	*EGFR*	*MMP9*	*EGFR*	*MMP9*	*EGFR*	*IL1B*
*EGFR*	*E2F1*	*IL10*	*BCL2*	*BCL2*	*BCL2*	*EGFR*	*BCL2*	
*TGFB1*	*BCL2*	*MYC*	*IL1B*	*TGFB1*	*IL10*	*IL10*	*IL1B*	*MMP9*
*IL10*	*IL10*	*ICAM1*	*SOX2*	*IL10*	*TGFB1*	*TGFB1*	*SOX2*	
*E2F1*	*CASP8*	*TGFB1*	*IL10*	*RHOA*	*MMP2*	*E2F1*	*RHOA*	*EGFR*
*MMP2*	*SOX2*	*MMP2*	*AKT2*	*MMP2*	*ICAM1*	*MMP2*	*CASP8*	
*ERBB2*	*IL1B*	*E2F1*	*RHOA*	*CASP8*	*ERBB2*	*ERBB2*	*PDCD4*	*TGFB1*
*CASP8*	*MYD88*	*ERBB2*	*MYD88*	*ERBB2*	*E2F1*	*ICAM1*	*RASA1*	
*RHOA*	*RHOA*	*FOXO3*	*CASP8*	*E2F1*	*CASP8*	*CASP8*	*AKT2*	*IL10*
*ICAM1*	*PDCD4*	*IGF1R*	*MMP2*	*IGF1R*	*RHOA*	*RHOA*	*MYD88*	
*FASLG*	*MAT2A*	*CASP8*	*E2F1*	*ICAM1*	*FASLG*	*CXCL10*	*IL10*	*E2F1*
*CXCL10*	*TGFBR2*	*CDK6*	*RASA1*	*FASLG*	*IGF1R*	*FASLG*	*E2F1*	
*FOXO3*	*RASA1*	*CXCL10*	*BMPR2*	*SOX2*	*CXCL10*	*FOXO3*	*ERBB2*	*CASP8*
*SOX2*	*AKT2*	*TLR2*	*PDCD4*	*FOXO3*	*FOXO3*	*IGF1R*	*MMP2*	
*MYD88*	*BMPR2*	*RHOA*	*ERBB2*	*CXCL10*	*TLR2*	*CDK6*	*BMPR2*	*RHOA*

The table shows the top 20 hub genes in the PPI network of hsa-miR-21 targets, ranked by eight CytoHubba algorithms (Degree, Bottleneck, MCC, Stress, Closeness, EPC, MNC, and Betweenness). The “Overlap” column lists genes consistently identified across all eight algorithms.

## Data Availability

The original contributions presented in this study are included in the article/Supplementary Materials. Further inquiries can be directed to the corresponding author.

## Supplementary Materials

The following supporting information can be downloaded at: https://www.mdpi.com/article/10.3390/ijms27020865/s1.
