# Supplementary material for: Integrative Bioinformatics Analysis of hsa-miR-21 in Breast Cancer Reveals a Prognostic Hub-Gene Signature
Source: Int J Mol Sci. 2026 Jan 15;27(2):865. doi: 10.3390/ijms27020865 (PMC12841244; doi:10.3390/ijms27020865)
Supplement: Supplementary file 1 [file ijms-27-00865-s001.zip › ijms-3943752-supplementary.pdf]

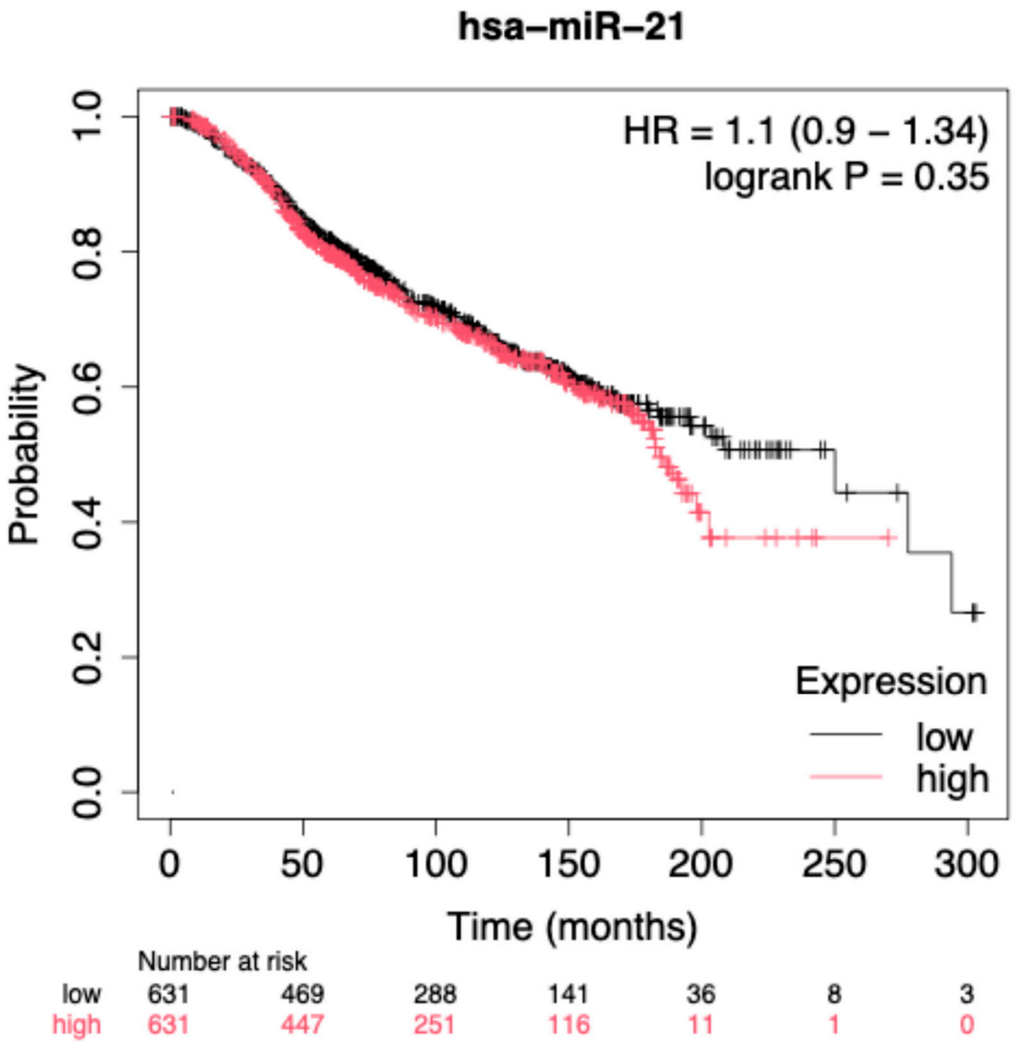

Figure S1. Kaplan–Meier overall survival analysis of hsa-miR-21 expression in the METABRIC breast cancer cohort.

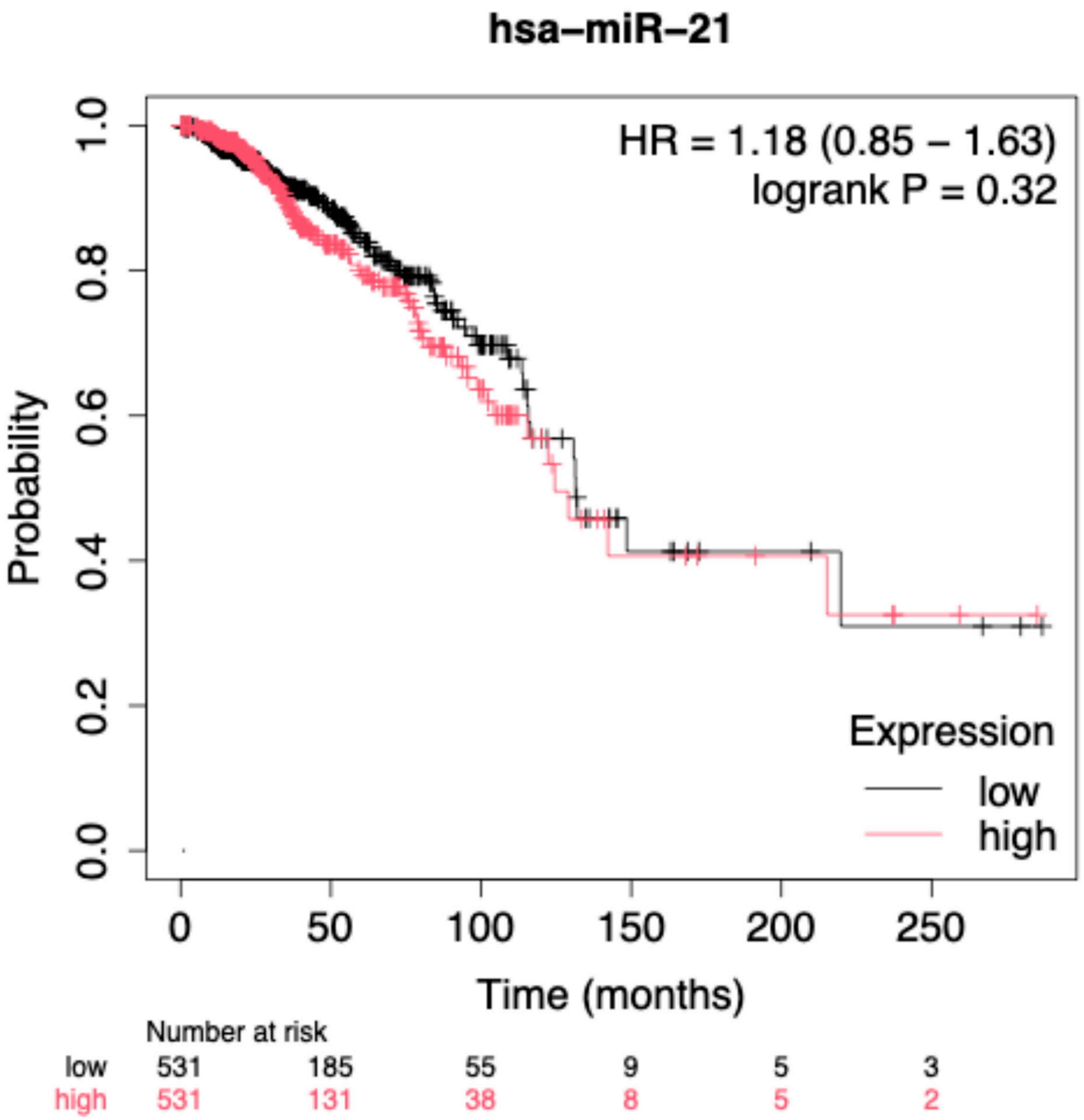

Figure S2. Kaplan–Meier overall survival analysis of hsa-miR-21 expression in the TCGA-BRCA cohort

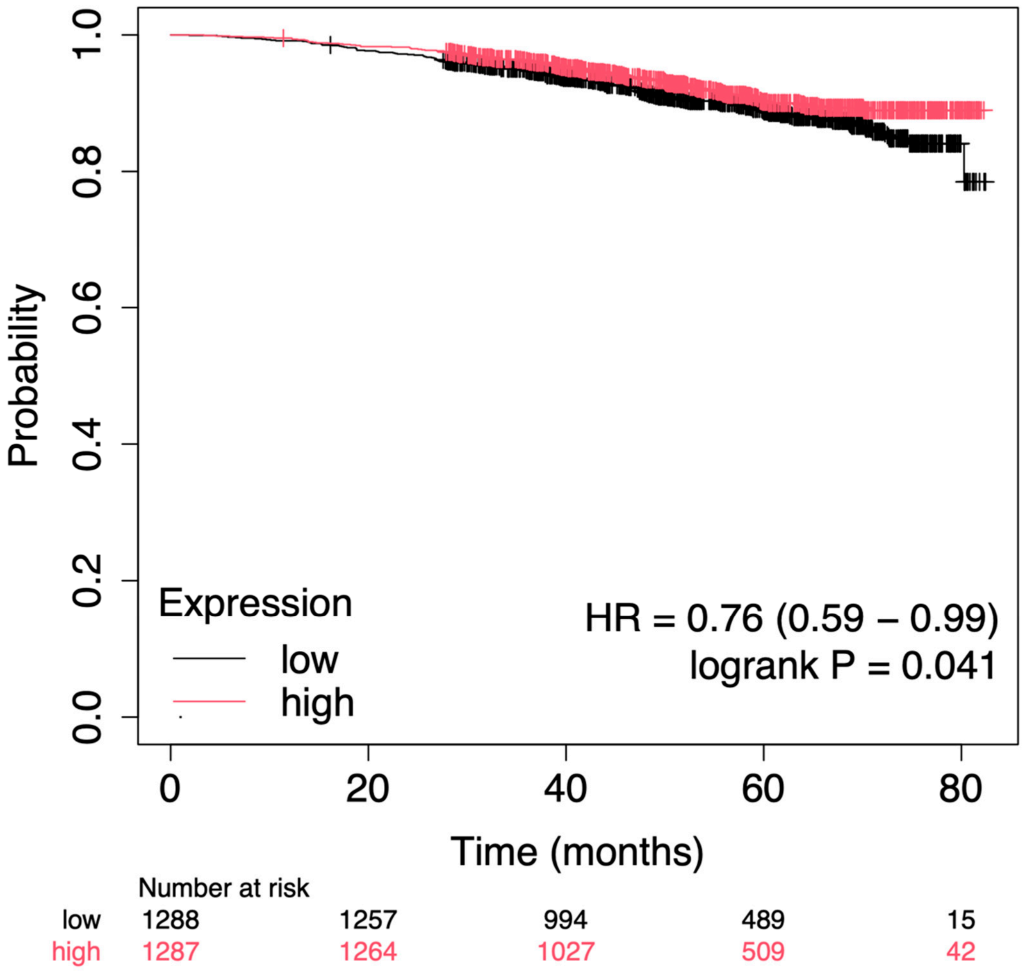

Figure S3. Kaplan–Meier overall survival analysis for the miR-21 hub-gene signature in ER-positive subgroup (n= 2.575).

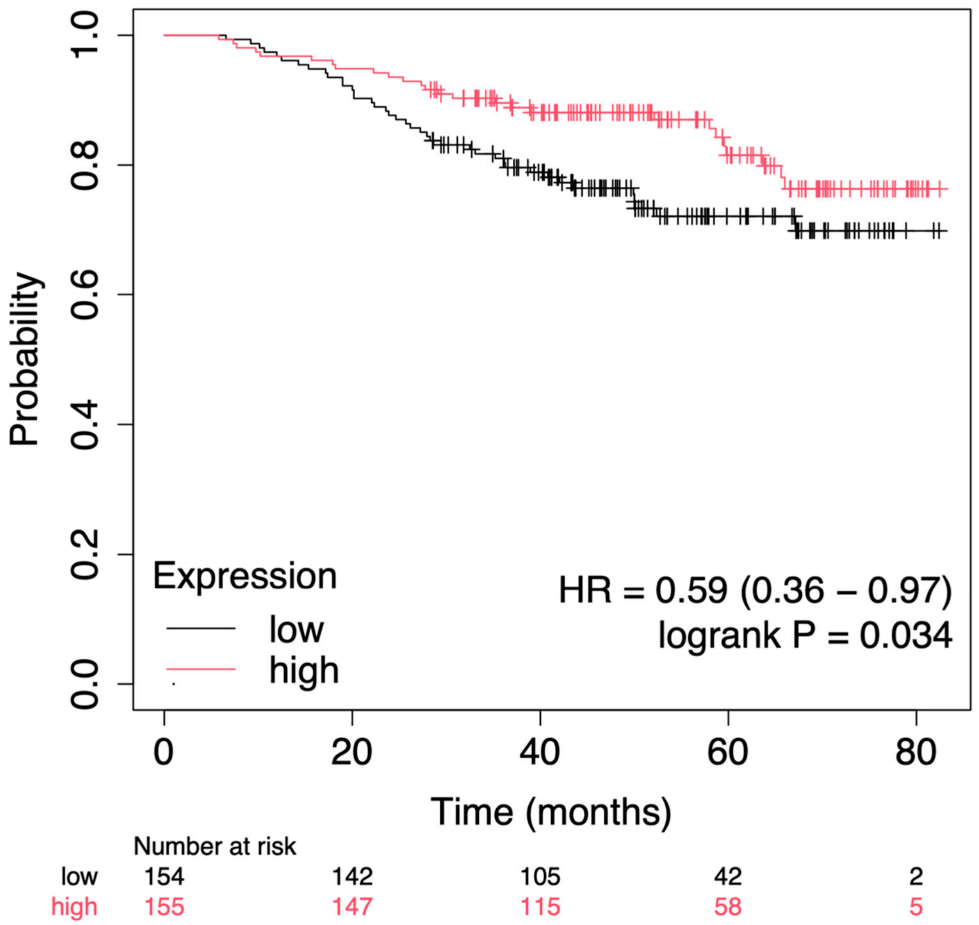

Figure S4. Kaplan–Meier overall survival analysis for the miR-21 hub-gene signature in Basal-like subgroup (n= 309).

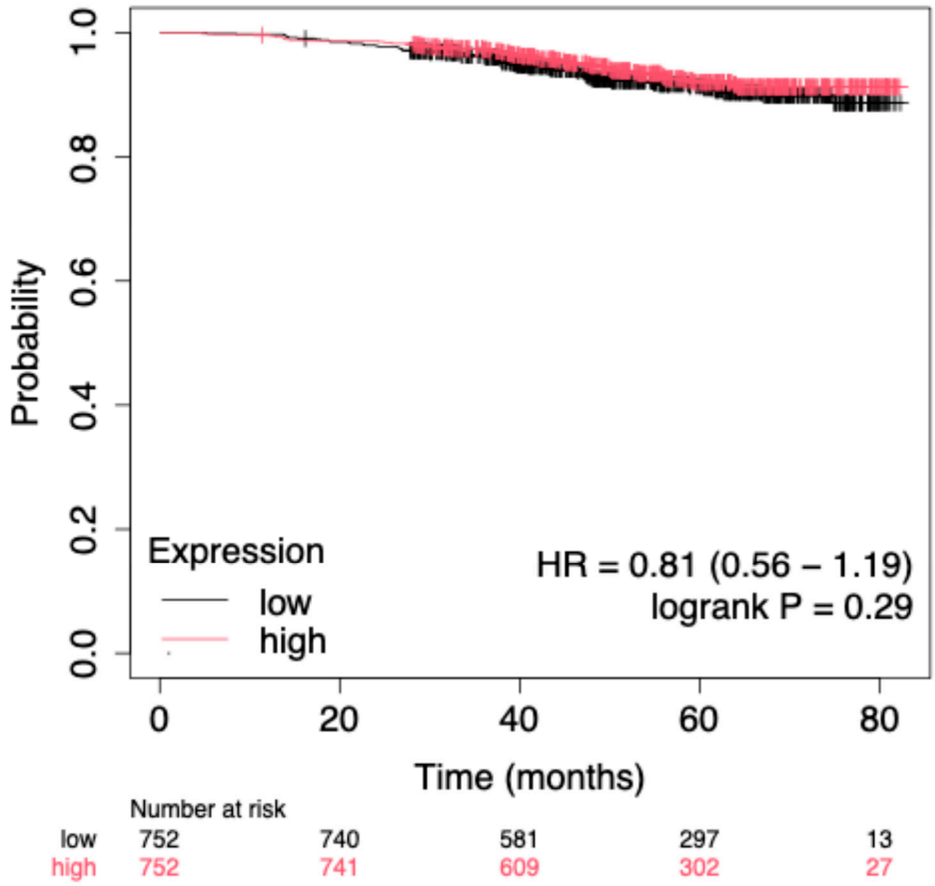

Figure S5. Kaplan–Meier overall survival analysis for the miR-21 hub-gene signature in Luminal A subgroup (n= 1.504).

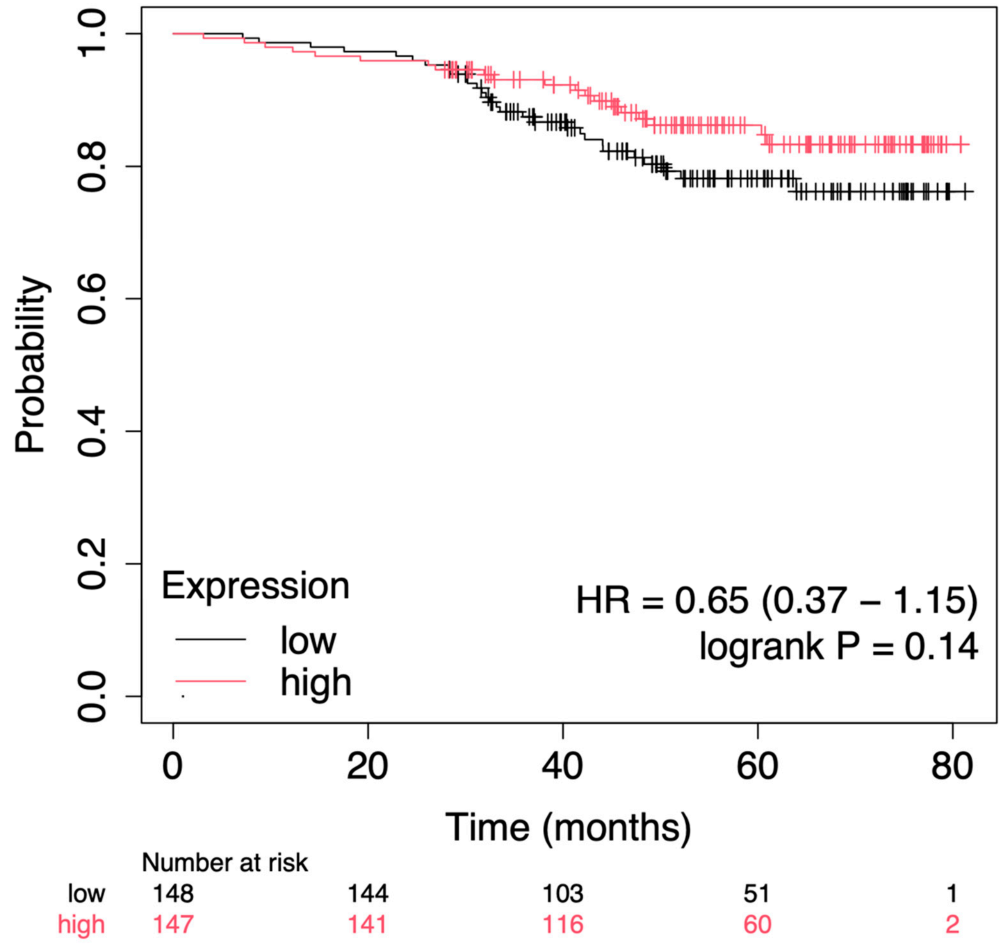

Figure S6. Kaplan–Meier overall survival analysis for the miR-21 hub-gene signature in HER2-enriched subgroup (n= 295).

Table S1: Significantly enriched GO terms of hub genes of miRNAs (GO terms are arranged according to their p-values, in descending order).

GO	GO ID	GENES	P-value	FDR
**Biological process**				
positive regulation of miRNA transcription	1902895	*MYC, EGFR, IL10, STAT3, TGFB1*	2,80E-08	2,00E-05
positive regulation of cell migration	0030335	*CASP8, EGFR, IL1B, RHOA, STAT3, TGFB1*	2,90E-07	1,00E-04
positive regulation of transcription by RNA polymerase II	0045944	*E2F1, MYC, EGFR, IL1B, IL10, PTEN, STAT3, TGFB1*	9,00E-07	2,10E-04
positive regulation of fibroblast proliferation	0048146	*E2F1, MYC, EGFR, IL1B*	2,90E-06	5,10E-04
positive regulation of cell population proliferation	0008284	*E2F1, MYC, EFGR, TGFB1, STAT3, PTEN*	5,00E-06	7,00E-04
positive regulation of canonical NF-kappaB signal transduction	0043123	*CASP8, IL1B, RHOA, STAT3, TGFB1*	6,10E-06	7,10E-04
cellular response to xenobiotic stimulus	0071466	*E2F1, MYC, EGFR, MYC*	7,80E-06	7,80E-04
cellular response to mechanical stimulus	0071260~	*CASP, EGFR, IL1B, TGFB1*	1,10E-05	9,40E-04
positive regulation of apoptotic process	0043065~	*BCL2, CASP8, E2F1, MMP9, TGFB1*	2,40E-05	1,80E-03
positive regulation of DNA-templated transcription	0045893	*E2F1, MYC, IL1B, IL10, STAT3, TGFB1*	2,50E-05	1,80E-03
**Molecular function**				
identical protein binding	0042802	*BLC2, MYC, CASP8, EGFR, MMP9, PTEN, STAT3, TGFB1*	1,30E-05	1,80E-03
DNA-binding transcription factor binding	0140297	*BCL2, E2F1, MYC, STAT3*	1,00E-04	6,50E-03
protein dimerization activity	0046983	*E2F1, MYC, IL10, STAT3*	1,40E-04	6,50E-03
cytokine activity	0005125	*IL1B, IL10, TGFB1*	5,20E-03	1,80E-01
ubiquitin protein ligase binding	0031625	*BCL2, CASP8, EGFR*	1,20E-02	3,40E-01
protein-containing complex binding	0044877	*MYC, CASP8, TGFB1*	1,60E-02	3,60E-01
enzyme binding	0019899	*EGFR, PTEN, TGFB1*	1,90E-02	3,60E-01
protein binding	0005515		2,30E-02	4,00E-01
protein kinase binding	0019901	*EGFR, RHOA, STAT3*	3,10E-02	4,70E-01
DNA-binding transcription factor activity	0003700	*EGFR, PTEN, TGFB1*	4,60E-02	6,00E-01
**Cellular component**				
protein-containing complex	0032991	*BCL2, E2FA, MYC, CASP8, EGFR*	3,30E-04	2,90E-02
nucleus	0005634	*BCL2, E2F1, MYC, CASP8, EGFR, PTEN, RHOA, STAT3, TGFB1*	4,30E-03	1,90E-01
extracellular space	0005615	*EGR, IL1B, IL10, MMP9, TGFB1*	1,30E-02	3,80E-01

cytoplasm	0005737	*BCL2, E2F1, MYC, CASP8, EGFR, PTEN, STAT3, TGFB1*	1,70E-02	3,90E-01
extracellular region	0005576	*IL1B, IL10, MMP9, PTEN, TGFB1*	2,60E-02	4,70E-01
secretory granule	0030141	*IL1B, TGFB1*	4,90E-02	5,80E-01
ruffle membrane	0032587	*EGFR, RHOA*	5,30E-02	5,80E-01
cytosol	0005829	*BCL2, CASP8, EGFR, IL1B, PTEN, RHOA, STAT3*	5,30E-02	5,80E-01
cytoplasmic side of plasma membrane	0009898	*PTEN, RHOA*	5,90E-02	5,80E-01
RNA polymerase II transcription regulator complex	0090575	*E2F1, STAT3*	6,50E-02	5,80E-01

Table S2: Significantly enriched KEGG pathway terms of hub genes of miRNAs (pathways are arranged according to their p-values, in descending order).

**KEGG pathway**	**KEGG ID**	**GENES**	**P-value**	FDR
Pathways in cancer	hsa05200	*BCL2, E2F1, MYC, CASP8, EGFR, MMP9, PTEN. RHOA, STAT3, TGFB1*	6,70E-10	6,00E-08
Hepatitis B	hsa05161	*BCL2, E2F1, MYC, CASP8, MMP9, STAT3, TGFB1*	1,90E-08	7,10E-07
MicroRNAs in cancer	hsa05206	*BCL2, E2F1, MYC, EGFR, MMP9, PTEN, RHOA, STAT3*	2,40E-08	7,10E-07
Breast cancer	hsa05224	*E2F1, MYC, EGFR, PTEN, STAT3, TGFB1, IL10*	1,30E-07	3,00E-06
Tuberculosis	hsa05152	*BCL2, CASP8, IL1B, IL10, RHOA, TGFB1*	1,70E-06	3,10E-05
Proteoglycans in cancer	hsa05205	*MYC, EGFR, MMP9, RHOA, STAT3, TGFB1*	3,10E-06	4,00E-05
Colorectal cancer	hsa05210	*BCL2, MYC, EGFR, RHOA, TGFB1*	3,10E-06	4,00E-05
Lipid and atherosclerosis	hsa05417	*BCL2, CASP8, IL1B, MMP9, RHOA, STAT3*	4,00E-06	4,60E-05
Prostate cancer	hsa05215	*BCL2, E2F1, EGFR, MMP9, PTEN*	5,10E-06	5,10E-05

Toxoplasmosis	hsa05145	*BCL2, CASP8, IL10, STAT3, TGFB1*	8,60E-06	7,80E-05
